# Structural validity of the Boston Carpal Tunnel Questionnaire and its short version, the 6-Item CTS symptoms scale: a Rasch analysis one year after surgery

**DOI:** 10.1186/s12891-020-03626-2

**Published:** 2020-09-12

**Authors:** Juhani Multanen, Jari Ylinen, Teemu Karjalainen, Joona Ikonen, Arja Häkkinen, Jussi P. Repo

**Affiliations:** 1 Department of Physical Medicine and Rehabilitation, Central Finland Central Hospital, Jyväskylä, Finland; 2 Faculty of Sport and Health Sciences, University of Jyväskylä, Jyväskylä, Finland; 3 Department of Surgery, Central Finland Central Hospital, Jyväskylä, Finland; 4 Department of Hand Surgery, Turku University Hospital and University of Turku, Turku, Finland

**Keywords:** Carpal Tunnel Release, Boston Carpal Tunnel Questionnaire, Six-Item Carpal Tunnel Symptoms Scale, Rasch analysis, Psychometrics

## Abstract

**Background:**

The Boston Carpal Tunnel Questionnaire (BCTQ) and its shorter version, the Six-Item Carpal Tunnel Symptoms Scale (CTS-6), are widely used for assessing function and/or symptoms in patients with carpal tunnel syndrome. This study examined the structural validity of the BCTQ and CTS-6 among patients who had undergone surgery for treatment of carpal tunnel syndrome.

**Methods:**

The data for this cross-sectional analysis were obtained from 217 adult patients who had undergone carpal tunnel release surgery 1 year earlier. All patients completed the CTS-6, Symptom Severity Scale (SSS) and Functional Status Scale (FSS) of the BCTQ at 12 months after surgery. The Rasch Measurement Theory (RMT) was applied to investigate the unidimensionality, residual correlation, differential item functioning, scale coverage/targeting, and person separation of the CTS-6, SSS and FSS of the BCTQ.

**Results:**

The FSS showed unidimensionality and good scale and item fit. All items showed ordered response category thresholds. Eight of the FSS items displayed differential item functioning favoring age or gender. The multidimensional structure of the CTS-6 was absorbed by creating a testlet for frequency of symptoms or testlets for pain and numbness. The testlets supported unidimensionality in the BCTQ SSS. One item in the CTS-6 and two items in the BCTQ SSS showed differential item functioning favoring age or gender. Four items in the BCTQ SSS and two items in the CTS-6 exhibited disordered response category thresholds. Merging of the relevant response categories led to ordered response category thresholds. The person separation indices were 0.73, 0.86 and 0.77 for the CTS-6, BCTQ SSS and FSS, respectively.

**Conclusions:**

Based on the RMT analysis, the CTS-6 has superior psychometric properties compared to the BCTQ SSS in surgically treated patients. The CTS-6 might be more accurate when separated into item sets measuring pain or numbness. The FSS of the BCTQ has acceptable construct validity, although gender differences at some ages were observed in responses.

## Background

The most common nerve entrapment is carpal tunnel syndrome (CTS) [1], which is caused by chronic pressure on the median nerve in the carpal tunnel of the wrist. Different etiological and risk factors for carpal tunnel syndrome have been described, including repetitive hand or wrist use, a high body mass index, tenosynovitis of the flexor tendons, and having a close relative with carpal tunnel syndrome [2–4]. The main symptoms are pain, numbness and tingling in the hand and arm. Carpal tunnel syndrome can cause functional deficits, typically weakness of palmar abduction or even complete inability to oppose the thumb. Rehabilitation using various maneuvers or night splinting of the wrist are used in mild cases, while carpal tunnel release (transection of the transverse carpal ligament) is an established treatment in more severe cases or when non-operative treatments fail to improve the symptoms [4].

Measurement of the treatment effect of carpal tunnel syndrome has shown an increasing shift towards the use of patient-reported outcomes (PROs) [5]. Condition-specific measures can provide valuable patient-centered information on the success of treatment [6]. The Boston Carpal Tunnel Questionnaire (BCTQ) PRO measure was developed in 1993 to assess symptoms and functional impairment caused by carpal tunnel syndrome [7]. The BCTQ has since been extensively tested using classical test theory and longitudinal validation methods [8–18]. It is assumed that a well-constructed PRO instrument designed to measure one latent trait will display a unidimensional structure. Unidimensionality means that the items in a questionnaire measure only a single underlying construct. This enables more accurate evaluation of longitudinal change in scores on the underlying trait. In clinical practice and research, it is also important that measures that adequately provide the requisite information are as short as possible. For these reasons, Atroshi and colleagues applied exploratory factor analysis and the Item Response Theory (IRT) to the BCTQ and produced a short version of the symptoms scale which they called the CTS-6 [19]. However, the construct validity of the BCTQ or CTS-6 has not been analyzed using the Rasch Measurement Theory (RMT).

RMT analysis provides information on the structural validity of the BCTQ and CTS-6. The RMT model allows investigation of the ability of a scale to measure a latent trait such as symptoms or function. Moreover, item and scale fit in the predefined model and responses to the construct-specific scales can be tested. Furthermore, response bias can be tested for each scale item using differential item functioning (DIF). DIF occurs when different groups within the sample, e.g., men and women, respond differently to an individual item. Whereas classical test theory and several other psychometric methods test how well a model fits the data, the RMT tests how well the data fit a predefined model. The RMT can thus be considered more robust and rigorous for psychometric analyses of construct validity.

This study applied the RMT model to the BCTQ and the CTS-6 to investigate their unidimensionality, item and scale fit, residual correlation, differential item functioning, scale coverage/targeting, and person separation in patients who had undergone carpal tunnel release due to carpal tunnel syndrome.

## Methods

### Participants

The study protocol was approved by the ethics committee of Central Finland Hospital District, Jyväskylä, Finland (record number 15U/2017). Adult patients with primary carpal tunnel syndrome who had undergone carpal tunnel release (NOMESCO procedure code ACC51 median nerve release) in the Department of Surgery were invited to participate in the study. During a one-year period, 528 patients underwent carpal tunnel release. These patients were contacted via regular mail at 1 year after surgery. Carpal tunnel syndrome had been diagnosed based on patient history, symptoms, clinical examination, and nerve conduction tests with electroneuromyography (ENMG) before traditional open carpal tunnel release. Patients who returned questionnaires with sufficient data and their written informed consent were included. The study protocol adhered to the Helsinki Declaration. Patients with insufficient PRO instrument data or inadequate comprehension of written and spoken Finnish were excluded from the analysis. According to the COSMIN guidelines, a sample size of at least 200 patients was required for the RMT analysis [20].

### PRO instruments

#### Boston Carpal Tunnel Questionnaire (BCTQ)

The BCTQ comprises two subscales. The Symptom Severity Scale yields PRO data on the level of symptoms, while the Functional Status Scale assesses the level of hand function. The Symptom Severity Scale consists of 11 items assessing pain, paresthesia, numbness, weakness, nocturnal symptoms, and difficulty of grasping. The Functional Status Scale contains eight items, which assess functional deficits in the following domains: writing, buttoning clothes, holding a book while reading, gripping a telephone handle, opening jars, performing household chores, carrying grocery bags, bathing and dressing. Each item is scored from 1 (no symptoms/difficulties) to 5 (the worst symptoms/cannot perform the activity at all). The mean score for each scale is calculated, resulting in a score between 1 and 5, with higher scores indicating worse symptoms or function. A Finnish version of the BCTQ, which was used in this study, has been previously validated [21].

#### The 6-item CTS symptoms scale (CTS-6)

The CTS-6 was derived, using exploratory factor analysis and Item Response Theory, from the BCTQ Symptom Severity Scale [19]. The scale contains 6 items on the severity and frequency of night and daytime numbness, tingling and pain. Each item is scored on a 5-point Likert scale from 1 (no symptoms) to 5 (most severe symptoms). The overall score for the scale is calculated as the mean of the answered items. One missing item is allowed. A Finnish version of our translated and cross-culturally adapted version of the original CTS-6 was used in this study (unpublished data).

### Statistical analysis

The RMT [22] was applied to investigate the unidimensionality, residual correlation, differential item functioning, scale coverage/targeting, and person separation of the scales. The analysis was based on statistical and illustrative tests. The RMT analytic technique is explained in more detail elsewhere [22–25].

Unidimensionality was tested using principal component analysis. Unidimensionality is one of the main assumptions of RMT and means that the items in a PRO instrument measure only a single construct (latent trait). Principal component analysis (PCA) was conducted to define the “Rasch factor”, e.g. the first factor identified with the highest eigenvalue. After identifying the Rasch factor, the existence of residual factors was examined by dividing the scale items into two groups according to their correlation coefficients with the second factor identified in the PCA. The items with a correlation coefficient above +0.3 formed one group and those with a correlation coefficient below -0.3 formed the other group. For each patient, person estimates for each item were calculated for both sets of items. A series of independent samples t-tests were conducted patient by patient and the estimates compared between the two item sets. A threshold of less than 5% of significant t-tests at the level of 0.05 was used to identify a unidimensional structure. We hypothesized that less than 5% of t-tests would be statistically significant. If the values exceeded this threshold, testlets were constructed based on the residual correlations of pairs of items [26]. A testlet can be defined as set (bundle) of items that have a common characteristic. These testlets are sometimes referred to as independent polychotomous super-items. A value +/- 0.2 was used to indicate residual correlation [27]. The partial credit model was then applied [28]. In the partial credit model, each item is ranked on a rating scale construct. Incorrect responses can provide partial credit for a correct response [28].

Fit statistics were used to estimate the fit of the data to the RMT model. Item-person interaction (log residuals), item-trait interactions (chi-square (χ2) values), and item characteristic curves were analyzed for item fit. χ2-values are used to investigate how well the difficulty of the item matches the ability of the respondent and hence the item’s ability to correctly discriminate different states of the measured trait. Fit residuals between -2.5 and +2.5 were considered acceptable. Values above or below this range may reflect over- or underdiscrimination in relation to the level of average discrimination and hence poor fit of the item to the RMT model and measurement inaccuracies. A high residual fit can provide information on the redundancy of a specific item, as the item may not contribute any new information to the measurement [25]. The authors hypothesized that there would be no statistically significant findings in item fit after Bonferroni adjustment.

Differential item functioning was examined across age and gender. Differential item functioning analysis provides information about potential response bias. Two types of differential item functioning can be distinguished. In cases of uniform differential item functioning, the difference in probability remains constant across different levels of symptoms. In non-uniform differential item functioning, the groups have different probabilities at different levels of symptoms. DIF for age was tested by dividing the age groups into two based on the mean age of the study population. DIF for gender was tested for groups of men and women. We hypothesized that the items would not show differential item functioning.

Person-item thresholds were examined to reveal how well the scale targets and covers patients who have undergone carpal tunnel release due to carpal tunnel syndrome. Person and item locations can be evaluated based on logits. The authors hypothesized no difference between the age or gender distributions. Statistical significance was set at 0.05.

Response category thresholds were investigated to ascertain how well the adjacent response categories function. A threshold indicates the point where there is a 50% probability that the response will fall into either one of the two adjacent categories. A disordered threshold curve indicates that the response categories are not functioning as intended. Possible reasons for this include confusing response category wording, or an inappropriate number of distinct response categories, meaning that respondents have difficulties in deciding which category to select. In an ordered threshold category, the illustrated pattern is symmetrically distributed across each of the response thresholds. We hypothesized that the response categories would be ordered. If violations of the pattern were found, merging the response categories in items with disordered response category thresholds would be trialed.

The person separation index was measured for reliability. It can be used to test whether the scale discriminates between patients varying in their health status (e.g. function or symptoms). A low person separation index value indicates that the scale might not be sensitive enough to discriminate between patients who have or do not have the disability in question. Values were hypothesized to be at least 0.7 (allows separation of 2 groups).

## Results

In total, 259 patients participated in the study (response rate 49%). Of these, 217 had provided adequate data and were included in the analysis. Patients’ sociodemographic and clinical data are presented in Table 1.

**Table 1:** Patients’ sociodemographic and clinical characteristics.

Variable	All, *N*=217
Female, n (%)	140 (65)
Age, mean (SD)	60 (14)
Body mass index, kg/m^2^, mean (SD)	28.4 (5.2)
Employment status, n (%)	
Employed	103 (48)
Unemployed	10 (4)
Retired / Pensioner	104 (48)
Current smokers, n (%)	27 (12)
Operated side, n (%)	
Right	87 (40)
Left	41 (19)
Bilateral	89 (41)
Duration of symptoms before operation, months, median (IQR)	18 (12, 36)
Pain, VAS range 0-100, mean (SD)	19.2 (22.9)
Pain medication, n (%)	35 (16)

*SD* standard deviation, *IQR* interquartile range

### CTS-6

The unidimensionality of the CTS-6 was not supported, as more than 5% of t-tests were statistically significant (Table 2). Seven of the 19 pairs of items had a residual correlation above 0.2. The residual correlation matrix is available in the additional file (see Additional file 1). No clear pattern for testlet creation based on the residual correlations was observed. A testlet was created based on items focusing on symptom frequency. This led to a unidimensional structure with less than 5% of statistically significant t-tests at 0.05 probability. The testlet (items 5 and 6) explained 35% of the total non-error variance. A second analysis with testlets based on items focusing on pain or numbness also led to a unidimensional structure with less than 5% of statistically significant t-tests at 0.05 probability. Testlet 1 (items 1, 2, 5) and testlet 2 (items 3, 4, 6) explained 97.3% and 100% of the total non-error variance, respectively.

**Table 2:** Fit statistics and unidimensionality of the scales

Subscale	Items		Persons		Chi square	DF	P	PSI (extremes/ no extremes)	Percentage (%) of significant t-tests
	Location (mean, SD)	Fit residual (mean, SD)	Location (mean, SD)	Fit residual (mean, SD)					
CTS-6	0.00 (0.69)	-0.14 (1.08)	-2.77 (1.83)	-0.45 (1.18)	15.2	18	0.65	0.73/0.78	5.2
Testlet 1	0.00 (0.75)	0.01 (1.11)	-2.70 (1.77)	-0.39 (1.12)	14.2	15	0.51	0.73/0.79	3.9
Testlet 2	0.00 (0.30)	0.09 (0.45)	-1.51 (1.03)	-0.48 (0.77)	8.5	6	0.20	0.54/0.63	2.0
Symptoms BCTQ	0.00 (0.91)	-0.42 (1.33)	-2.56 (1.99)	-0.42 (1.19)	58.4	33	0.004	0.86/0.88	18.1
Testlets	0.00 (0.10)	-0.10 (0.73)	-0.92 (0.83)	-0.46 (0.74)	18.0	6	0.006	0.86/0.88	2.1
Function BCTQ	0.00 (1.31)	-0.18 (1.10)	-3.51 (1.84)	-0.28 (0.97)	34.8	24	0.07	0.77/0.83	2.5

*DF* degrees of freedom, *P* P-value, *PSI* person separation index, *SD* standard deviation, *CTS-6* The 6-item CTS symptoms scale, *BCTQ* Boston Carpal Tunnel Questionnaire

**Fig. 1:**
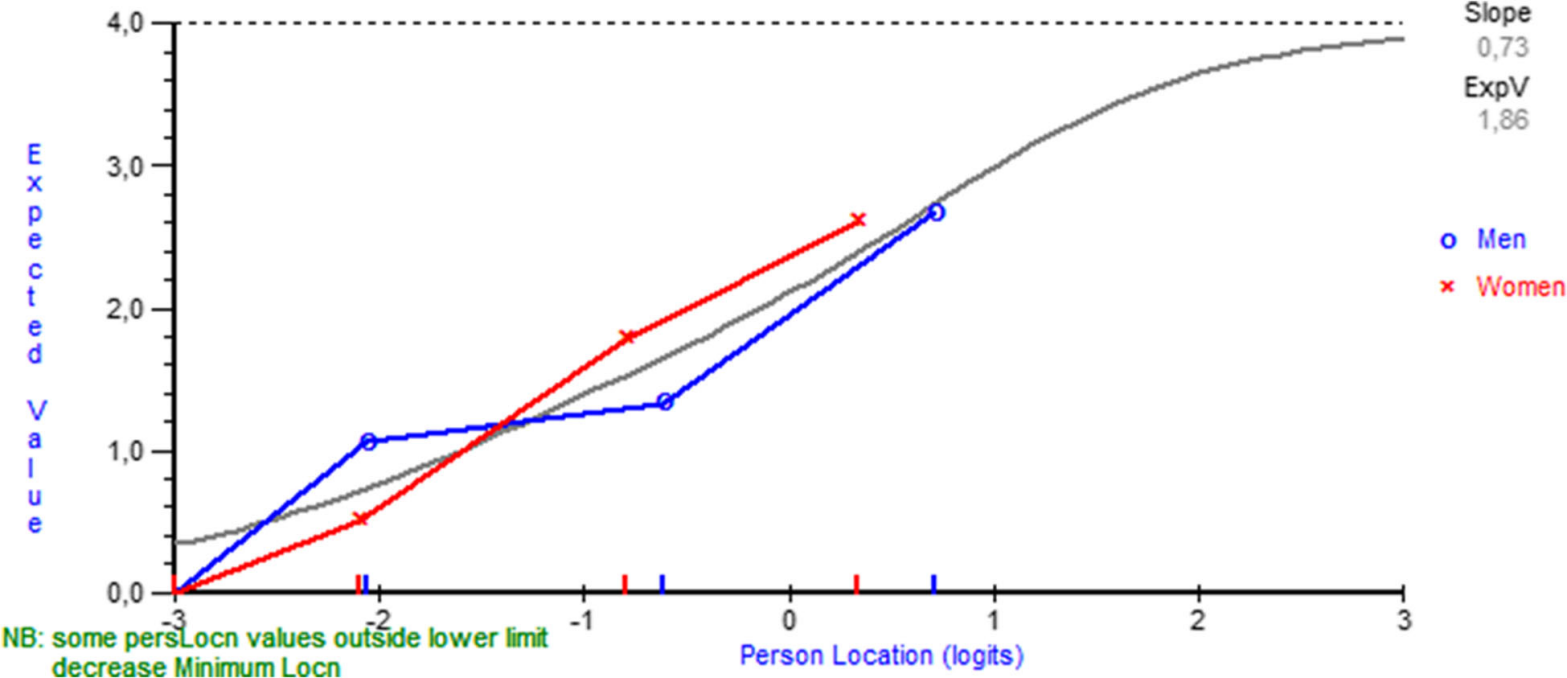
Differential item functioning by gender for item 6 (“How often did the following symptoms in your hand wake you up at night? - numbness or tingling”) of the CTS-6. Note in figure: ‘Some person location values … decrease minimum location’ and ‘Person location’

All the CTS-6 items had fit residuals within the -/+2.5 range, indicating acceptable item fit (Table 3). After Bonferroni correction, item 6 showed non-uniform differential item functioning favoring gender over age (P<0.001) (Fig. 1).

**Table 3:** Item fit statistics and differential item functioning (DIF) for age and gender

**CTS-6**	**Location**	**Fit residual**	**DF**	**Chi-square**	**P**	**DIF Gender (U/NON-U)**	**DIF Age (U/NON-U)**
**Item**							
How severe are the following symptoms in your hand?							
1. Pain at night	0,068	-1,212	123,67	4,006	0,26	-	-
2. Pain during daytime	1,209	1,671	123,67	1,773	0,62	-	-
3. Numbness or tingling at night	-0,752	-0,524	123,67	1,749	0,62	-	P=0.0498
4. Numbness or tingling during daytime	-0,441	0,520	123,67	3,048	0,38	-	-
How often did the following symptoms in your hand wake you up at night?							
5. Pain	0,239	-0,220	123,67	1,381	0,71	-	-
6. Numbness or tingling	-0,323	-1,057	123,67	3,249	0,36	(P=0.0008)	-
**BCTQ Symptom Severity Scale**	**Location**	**Fit residual**	**DF**	**Chi-square**	**P**	**DIF Gender (U/NON-U)**	**DIF Age (U/NON-U)**
**Item**							
1. How severe is the hand or wrist pain that you have at night?	0,598	-0,879	167,00	1,893	0,60	-	-
2. How often did hand or wrist pain wake you up during a typical night in the past two weeks?	2,133	1,745	167,00	3,011	0,39	-	-
3. Do you typically have pain in your hand or wrist during the daytime?	0,373	-2,112	167,00	7,312	0,06	-	-
4. How often do you have hand or wrist pain during daytime?	-0,78	-0,929	167,00	6,736	0,08	-	-
5. How long on average does an episode of pain last during the daytime?	-1,112	-0,232	167,00	9,340	0,03	-	NON-U; P=0.020
6. Do you have numbness (loss of sensation) in your hand?	-0,290	1,089	167,00	9,340	0,13	-	-
7. Do you have weakness in your hand or wrist?	-0,915	1,507	167,00	7,979	0,05	U; P=0.003	-
8. Do you have tingling sensations in your hand?	-0,141	-0,648	167,00	1,421	0,70	-	-
9. How severe is numbness (loss of sensation) or tingling at night?	-0,007	-1,604	167,00	4,850	0,18	-	-
10. How often did hand numbness or tingling wake you up during a typical night during the past two weeks?	0,510	-0,917	167,00	3,590	0,31	-	-
11. Do you have difficulty with the grasping and use of small objects such as keys or pens?	-0,369	1,818	167,00	6,739	0,08	-	-
**BCTQ Functional Status Scale**	**Location**	**Fit residual**	**DF**	**Chi-square**	**P**	**DIF Gender (U/NON-U)**	**DIF Age (U/NON-U)**
**Item**							
1. Writing	1,454	0,140	137,88	6,934	0,07	-	
2. Buttoning of clothes	-0,591	-0,188	137,88	2,659	0,45	U; P=0.020	U; P<0.001
3. Holding a book while reading	-0,997	0,985	137,88	2,626	0,45	-	-
4. Gripping of a telephone handle	1,462	-0,542	137,88	3,175	0,37	NON-U; P=0.0007	U; P=0.0002
5. Opening of jars	-1,623	1,471	137,88	3,585	0,31	-	U; P<0.001
6. Household chores	-0,477	-1,555	137,88	6,488	0,09	NON-U; P=0.020	-
7. Carrying of grocery bags	-0,887	-0,094	137,88	2,540	0,47	-	U; P=0.004
8. Bathing and dressing	1,659	-1,660	137,88	6,785	0,08	-	U; P=0.020

*CTS-6* The 6-item CTS symptoms scale, *DF* degrees of freedom, *P* P-value, *DIF* differential item functioning, *U* uniform, *NON-U* non-uniform, *BCTQ* Boston Carpal Tunnel Questionnaire

**Fig. 2:**
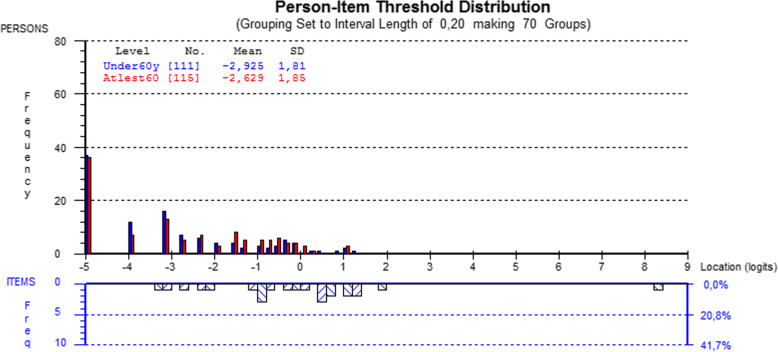
Person-item threshold distribution map for the CTS-6. Note in figure: Grouping set to interval length of 0,20, making 70 groups ‘Under 60yrs’ ‘At least 60yrs’

The scale covered patients located between -3 and 2 logits (Fig. 2). No statistically significant age difference was observed between the person-item threshold distributions (p = 0.23). However, a gender difference was found between the person-item threshold distributions (p = 0.03).

**Fig. 3:**
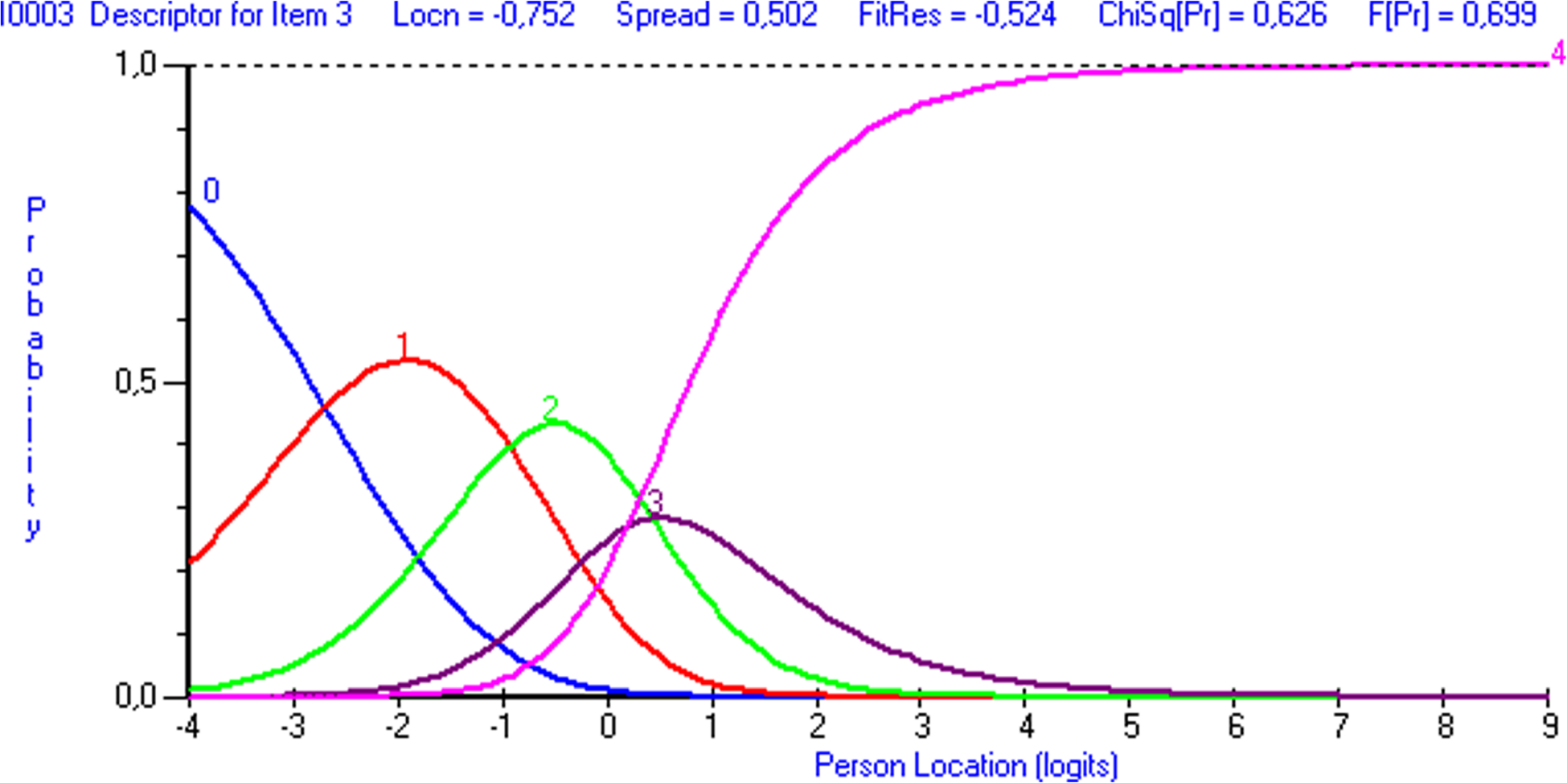
Response category threshold curve showing disordered thresholds for item 3 (“How severe are the following symptoms in your hand? –numbness or tingling at night”) in the CTS-6. The response options were 0= “None”, 1= “Mild”, 2= “Moderate”, 3= “Severe”, 4= “Very severe”. Note in figure ‘Person location’

**Fig. 4:**
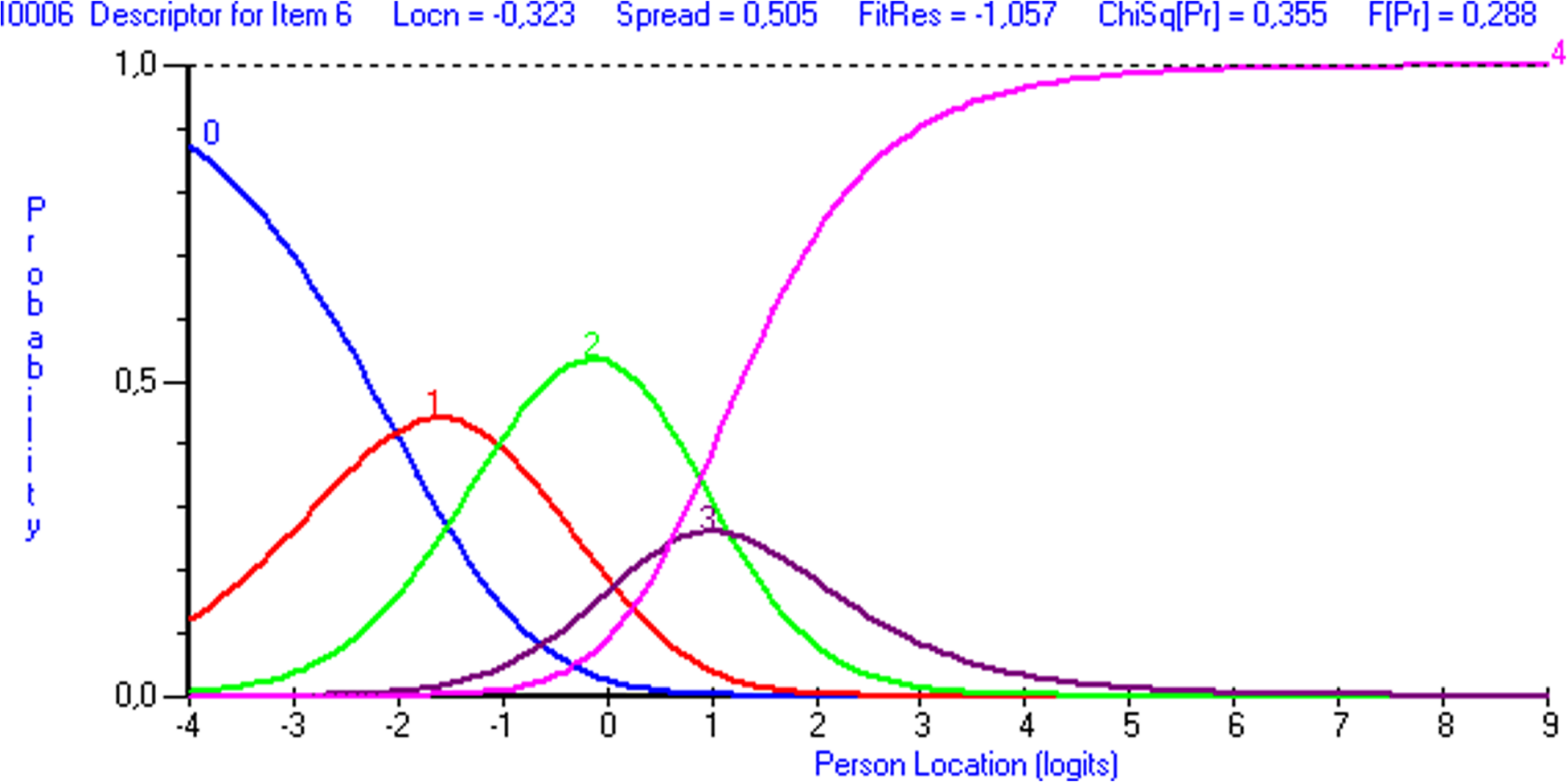
Response category threshold curve showing disordered thresholds for item 6 (“How often did the following symptoms in your hand wake you up at night? –numbness or tingling”) in the CTS-6. The response options were 0= “Never”, 1= “Once”, 2= “2 or 3 times”, 3= “4 or 5 times”, 4=“More than 5 times”. Note in figure ‘Person location’

**Fig. 5:**
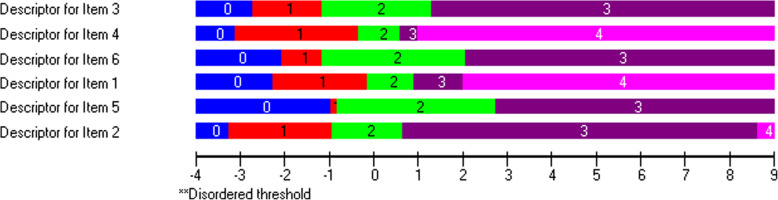
Response category threshold map showing ordered thresholds after merging the response categories of items 3 and 6 in the CTS-6

Items 3 and 6 showed disordered thresholds (Figs. 3 and 4). After trying out different options to merge the response categories, the best results were achieved by merging response categories 2 (“Moderate/2 or 3 times”) and 3 (“Severe/4 or 5 times”) with items 3 and 6, respectively. This led to disordered response category thresholds in item 5. Merging response categories 2 and 3 led to ordered thresholds in each item (Fig. 5). Note that categories 0-4 in this results section correspond to categories 1-5 in the CTS-6 and BCTQ (0=1, 1=2, 2=3, 3=4, 4=5).

### BCTQ Symptom Severity Scale

The analysis did not support unidimensionality. A residual correlation over 0.2 (range -0.531 to -0.214 and 0.239 to 0.650) was found in 34 of the 56 pairs of items (Additional file 1). Based on the residual correlation and clinical relevance, two testlets were created. The first testlet comprised the five items on pain (items 1-5) and the other testlet the remaining six items on numbness, tingling, weakness, or fine motor skills of the hand (items 6-11). The two testlets absorbed the multidimensional structure with 2.1% of t-tests significant at 0.05 probability. Testlet 1 (items 1-5) and testlet 2 (items 6-11) explained 96.2% and 100% of the total non-error variance, respectively.

All items in the Symptom Severity Scale had fit residuals within the -/+2.5 range, indicating acceptable item fit.

**Fig. 6:**
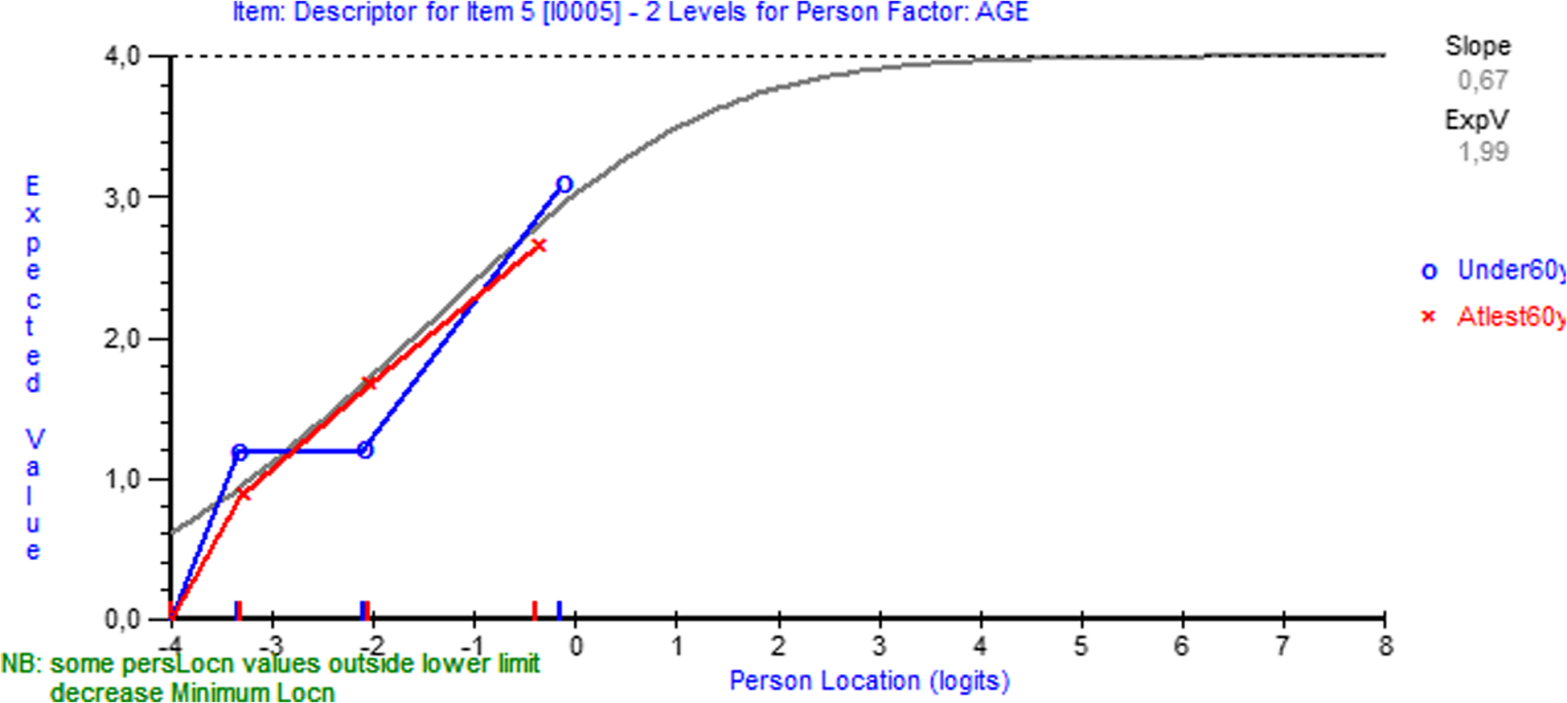
Differential item functioning favoring age in item 5 (“How long on average does an episode of pain last during the daytime?”) of BCTQ Symptom Severity Scale. Note in figure: ‘Some person location values … decrease minimum location’ and ‘Person location’

**Fig. 7:**
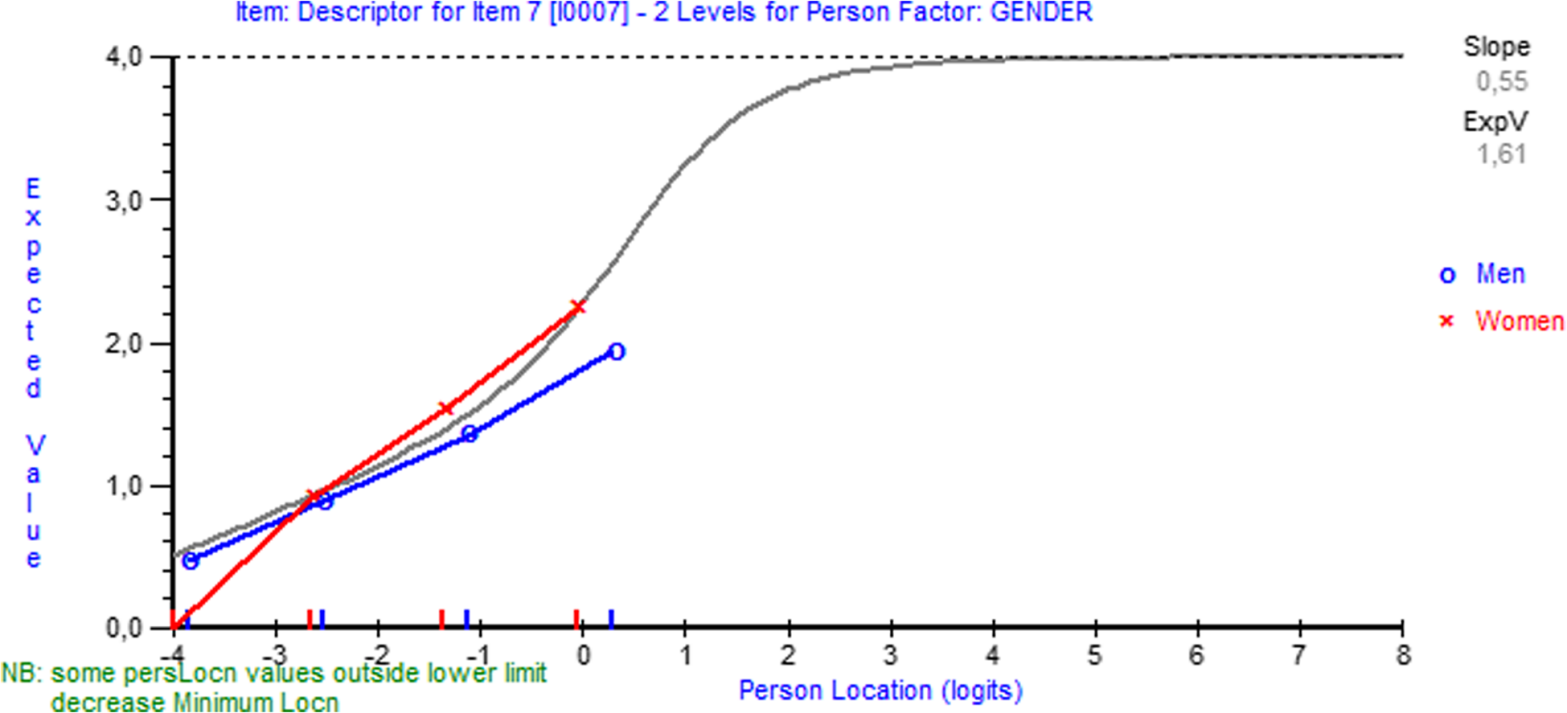
Differential item functioning by gender in item 7 (“Do you have weakness in your hand or wrist?”) of the BCTQ Symptom Severity Scale. Note in figure: ‘Some person location values … decrease minimum location’ and ‘Person location’

Item 5 showed non-uniform differential item functioning favoring age (p=0.02) (Fig. 6), and item 7 uniform differential item functioning favoring gender (p=0.003) (Fig. 7).

**Fig. 8:**
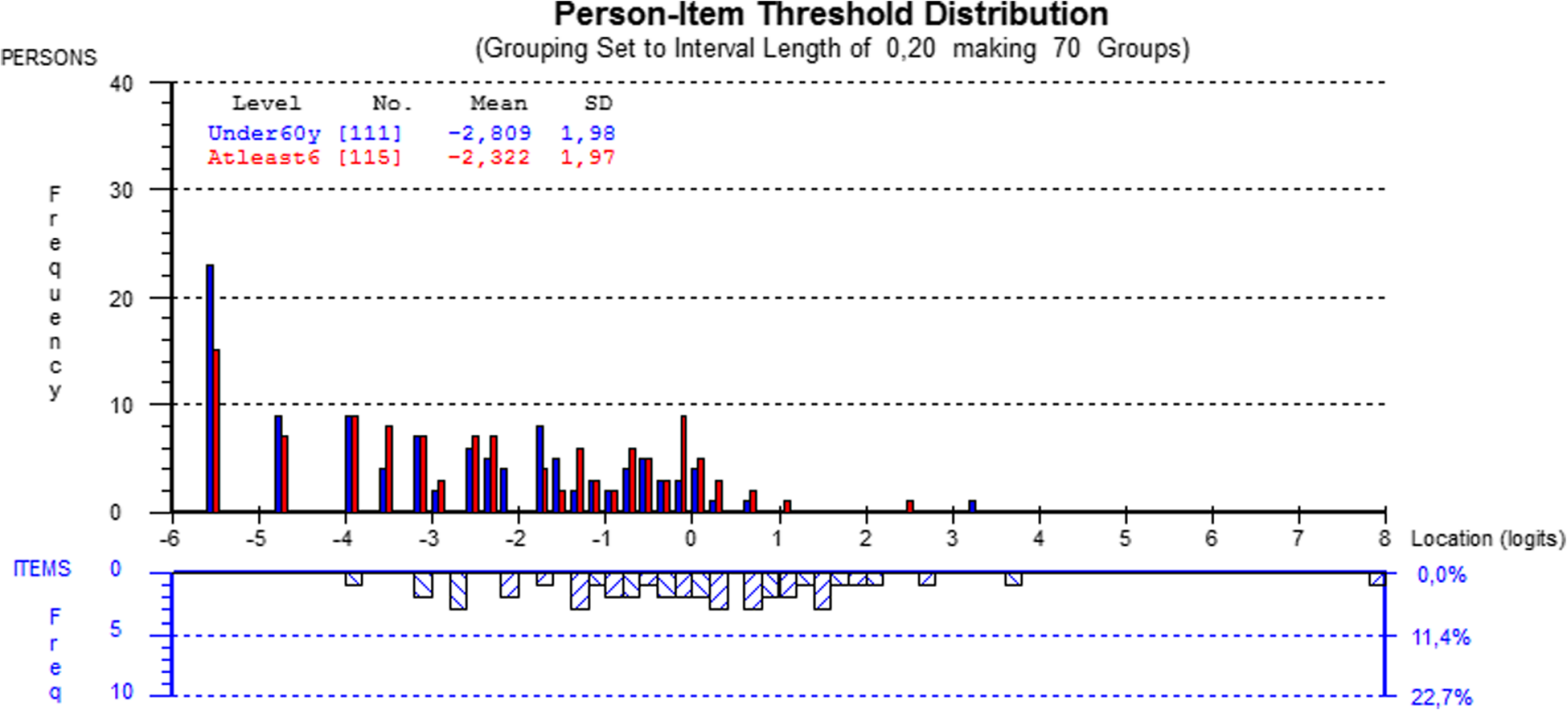
Person-item threshold distribution map for the BCTQ Symptom Severity Scale. Note in figure: Grouping set to interval length of 0,20, making groups’ ‘Under 60yrs’ ‘At least 60yrs’

The scale covered patients located between -4 and 4 logits. No difference was observed in the person-item threshold distribution by gender (p=0.50) or age (p=0.07) (Fig. 8).

**Fig. 9:**
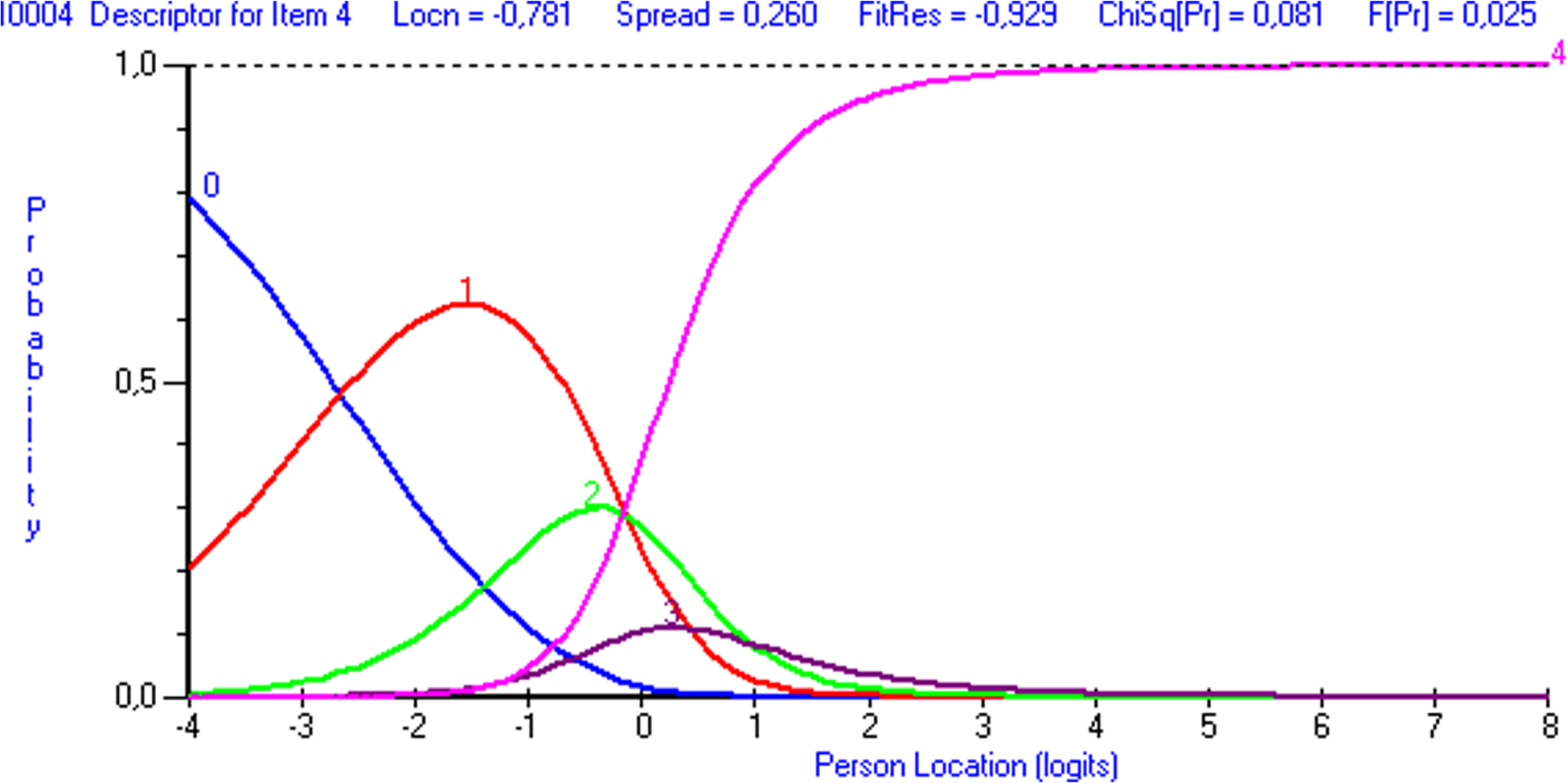
Response category threshold curve showing disordered thresholds for item 4 (“How often do you have hand or wrist pain during daytime?”) in the BCTQ Symptom Severity Scale. The response options were 0= “Normal”, 1= “1-2 times / day”, 2= “3-5 times / day”, 3= “More than 5 times”, 4= “Continuous”. Note in figure: ‘Person location’

**Fig. 10:**
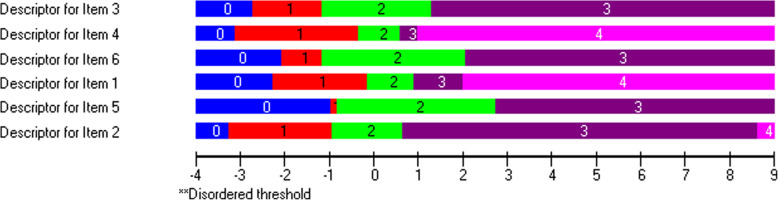
Response category threshold map showing ordered thresholds after merging the response categories in items 4 (“How often do you have hand or wrist pain during daytime?”), 5 (“How long on average does an episode of pain last during the daytime?”), 7 (“Do you have weakness in your hand or wrist?”) and 11 (“Do you have difficulty with the grasping and use of small objects such as keys or pens?”) of the BCTQ Symptom Severity Scale

Items 4, 5, 7 and 11 showed disordered thresholds (Fig. 9). Merging response categories 2 and 3 led to ordered thresholds adhering to the Guttman pattern, according to which if a patient can successfully answer an item of a certain level of difficulty, the patient would also be able to answer earlier items of lesser difficulty (Fig. 10).

### BCTQ Functional Status Scale

The Functional Status Scale showed unidimensionality. The proportion of statistically significant t-tests at 0.05 probability was 2.5%.

The scale showed good scale and item fit (Table 3).

A residual correlation was found between 12 of the 34 pairs of items (Appendix 1).

Three items (2, 4, 6) showed differential item functioning favoring gender and five (2, 4, 5, 7, 8) favoring age (Table 3).

**Fig. 11:**
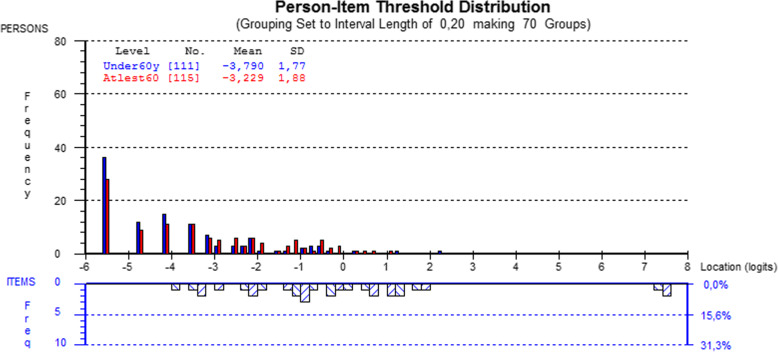
Person-item threshold distribution map for the BCTQ Functional Status Scale. Note in figure: Grouping set to interval length of 0,20, making groups’ ‘Under 60yrs’ ‘At least 60yrs’

The scale covered patients located between -4 and 2 logits. A slight difference was observed in the person-item threshold distribution by age (p=0.02) (Fig. 11).

No gender difference in thresholds was observed (p=0.98). All items had ordered threshold categories.

**Fig. 12:**
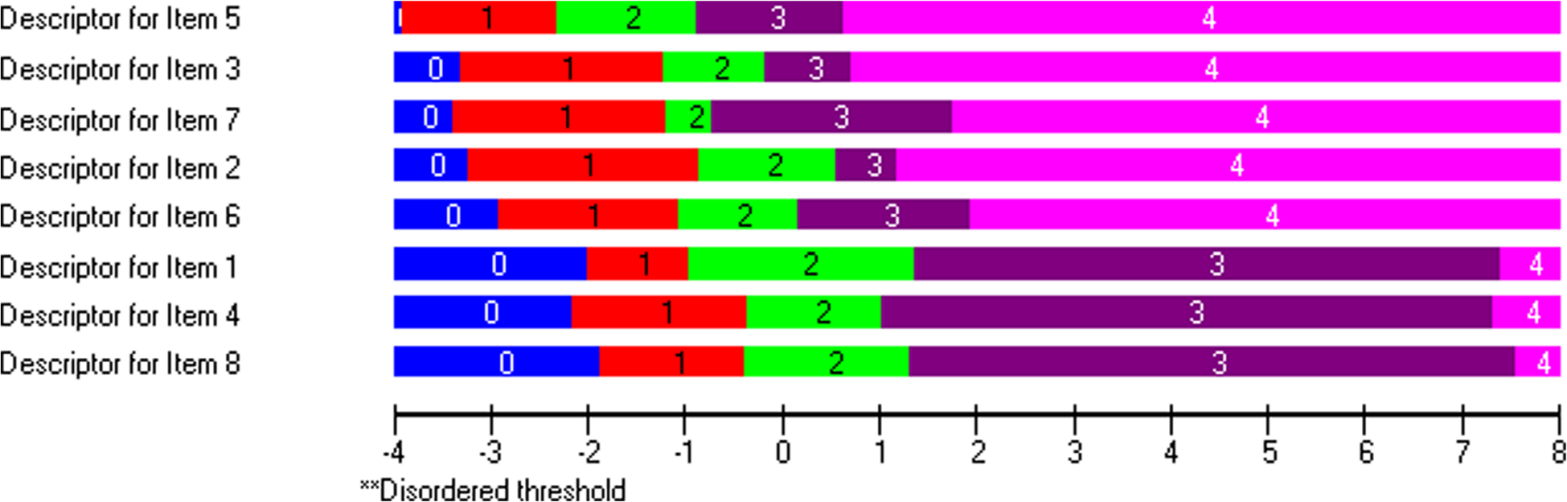
Response category threshold map showing ordered thresholds in the BCTQ Functional Status Scale

All eight items showed ordered thresholds (Fig. 12).

## Discussion

Rasch Measurement Theory analysis of the CTS-6 demonstrated a unidimensional structure after some adjustments, acceptable item fit to the model, a few disordered item thresholds and one significant differential item functioning favoring gender. The BCTQ Symptom Severity Scale in turn demonstrated multidimensionality, acceptable item fit to the RMT model, several disordered item thresholds and significant differential item functioning favoring gender and age. The BCTQ Functional Status Scale demonstrated a unidimensional structure, acceptable item fit, and several differential item functioning items favoring gender and age.

In this study, the CTS-6 was not found to have latent trait unidimensionality, raising concern as to whether the resulting score is valid when all items are summed. To solve the problem of multidimensionality, the non-fitting items can either be removed or new testlets created. While the CTS-6 showed good item fit (see below), the creation of a testlet for frequency of symptoms or testlets for pain and numbness were needed to solve the problem. Although the testlet for the frequency of symptoms demonstrated a unidimensional structure, two other testlets for pain and numbness were created, as these are clinically important symptoms and relevant to patients with carpal tunnel syndrome. The items in the testlets for pain and numbness were in a logical clinical relation to each other, and well suited to the formation of two separate subscales. Since both testlets satisfied the assumptions of the unidimensionality, the results of the study indicate that the CTS-6 might perform well when separated into item sets measuring pain or numbness (i.e., items 1, 2, 5 for pain and items 3, 4, 6 for numbness) in patients who have undergone carpal tunnel release.

We could not compare our results with previous studies because of the paucity of CTS-6 structural validity studies using the RMT model in patients with carpal tunnel syndrome. However, Atroshi and colleagues [19], who developed the CTS-6 from the longer version of the BCTQ Symptom Severity Scale by using exploratory factor analysis and IRT, found that in the patients undergoing carpal tunnel release, one dominant factor explained 58% of the variance, and that all 6 items were associated with that factor. Thus, they reasoned that the CTS-6 is unidimensional, as the first factor should be dominant and account for more than 20% of the variability. The difference between our result and that of Atroshi et al. may be explained by the different statistical methods used and patient groups studied (non-operated vs. operated).

In this study, unidimensionality was supported when the items of the BCTQ Symptom Severity Scale were bundled into two polytomous super-items. The BCTQ Functional Status Scale, however, demonstrated unidimensionality, indicating that all eight items in the scale measure the level of hand-related disability.

Item fit to the model was indicated to be good in the CTS-6 as well as in both BCTQ scales. In addition to item fit, person fit was also good at the questionnaire and testlet levels. This may partially be explained by the fact that the present sample of patients studied 1 year after carpal tunnel release surgery was rather homogeneous.

In the CTS-6, only item 6 (“How often did the following symptoms in your hand wake you up at night? - numbness or tingling”) exhibited significant non-uniform differential item functioning favoring gender after Bonferroni correction. The results suggested that women with mild symptoms tend to report waking up more often than male counterparts, whereas men with moderate to severe symptoms tend to report to wake up more often than female counterparts.

One item (“How long on average does an episode of pain last during the daytime?”) on the BCTQ Symptom Severity Scale exhibited non-uniform differential item functioning favoring age. This suggests that subjects under age 60 with moderate symptoms in the middle of the curve (Fig. 6) tended to report longer pain duration than those aged 60 or older. The BCTQ Symptom Severity Scale also contained one uniform differential item functioning favoring gender, viz. item 7 (“Do you have weakness in your hand or wrist?”). This suggests that, on the same level of symptoms, men tended to report more weakness in their hand or wrist than women. This is in line with Atroshi et al. [19] who found significant differential item functioning in item 7, with men showing higher values than women in patients undergoing carpal tunnel release. In the BCTQ Functional Status Scale several significant uniform and non-uniform differential item functioning items favoring gender and age were found, and hence the scale may give biased estimates of hand-related disability at different ages in both women and men. Taken together, the items of the CTS-6 and BCTQ Symptom Severity Scale showed that they are relatively invariant in both genders at different ages, whereas the BCTQ Functional Status Scale contains several invariant items. The BCTQ Functional Status Scale, in particular, could be improved by changing or deleting items in order to generate a differential item functioning-free scale for subjects who have undergone carpal tunnel release.

In their targeting ability, both the CTS-6 and BCTQ Symptom Severity Scale covered patients, as their mean obtained location score was around zero. This indicates a well-targeted measure which is neither too easy nor too hard. A positive mean location score would indicate that the sample as a whole was located at a higher level of symptoms than the mean, while a negative value would indicate the opposite. The results of the CTS-6 and BCTQ Symptom Severity Scale also revealed no differences between the age or gender distributions in the person-item threshold. Instead, the mean location score for the BCTQ Functional Status Scale was negative, indicating that the sample as a whole was located at a lower level of disability than the mean. In practice, this means that the BCTQ Functional Status Scale may have limited ability to detect functional status, or changes in it, in patients who have undergone carpal tunnel release and thus already have lower disability. This may especially be the case in younger subjects, as in this study the patients under age 60 exhibited lower disability values than those aged 60 above. No gender difference was noted in the person-item threshold distribution.

In the RMT model, the person separation index is used instead of reliability indices. However, the person separation index is analogous to a reliability index. The separation index describes the ratio of genuine separation to separation including measurement error. In the present study, the person separation index values of 0.73, 0.86 and 0.77 for the CTS-6, BCTQ Symptom Severity and Functional Status Scales, respectively, demonstrated good reliability for all three measures, as a minimum value of 0.7 is required for group use and 0.85 for individual use [29]. These reliability values are on more or less the same level as observed by Atroshi et al. [19] In their study, the reliability of the person separation index was 0.87 for the Symptom Severity Scale, and 0.88 for the CTS-6, indicating that the performance of the CTS-6 is similar to that of the original 11-item Symptom Severity Scale. In our recent report on a test-retest reliability experiment with most of the same subjects (N=193), we showed, from the perspective of classical test theory, that both of the BCTQ scales had high internal consistency, with a Cronbach’s alpha of 0.93 for both symptoms and function [21]. In the RMT partial credit model analysis, the calculation is equivalent to the Cronbach’s alpha, except that the logit value, as opposed to raw score, is used in the same formula. Both reports, with slightly different sample sizes, confirm that the scales perform well in separating respondents on the latent trait continuum.

Item threshold analysis may show a disordered threshold, if the response option wording is ambiguous, or if respondents find it difficult to discriminate between response options. In the present item threshold analysis of the CTS-6, items 3 (“How severe are the following symptoms in your hand? - numbness or tingling at night”) and 6 (“How often did the following symptoms in your hand wake you up at night? - numbness or tingling”) were disordered. In both items, merging response categories 3 (moderate/2 or 3 times) and 4 (severe/4 or 5 times) in the 5-point scale resulted in disordered response category thresholds in item 5 (“How often did the following symptoms in your hand wake you up at night? –pain”). However, merging response categories 3 and 4 in the 5-point scale produced ordered thresholds for each item and made the response categories work as intended. These results suggest that response options 3 and 4 in certain original items of the CTS-6 may be semantically or numerically too close to one another.

In the item threshold analysis of the BCTQ Symptom Severity Scale, 7 of the 11 items showed ordered categories. In the 4 items which showed disordered thresholds (items 4, 5, 7 and 11), merging response categories 3 (≈ moderate) and 4 (≈ severe) in the 5-point scale produced ordered thresholds. All eight items in the BCTQ Functional Status Scale had ordered thresholds, indicating that patients were able to differentiate between the response options.

The main strength of this study is that it is the first to systematically investigate the structural validity of the BCTQ and 6-item CTS by applying the RMT Model. We also had a sample of well over 200 patients, which may be regarded as of sufficient size for conducting psychometric analyses [20].

A limitation is that we only had patients who had undergone surgery for treatment of carpal tunnel syndrome. Thus, the results are generalizable mainly to patients in that situation. Another limitation relates to the fact that the questionnaires were distributed to individuals 1 year after surgery. It is thus possible that many symptoms will have resolved by this time point. The third limitation concerns the cross-sectional study design, which does not allow the monitoring of the test-retest reliability and responsiveness of the questionnaires. We have, however, previously measured and reported on the test-retest reliability of the BCTQ (Symptom Severity Scale and Functional Status Scale) with the most of the same patients [21]. Both scales showed excellent test-retest reliability, each with an intraclass correlation coefficient greater than 0.8. Nevertheless, the responsiveness of the BCTQ and CTS-6 remains to be studied in heterogeneously treated carpal tunnel syndrome patients. To the best of the authors’ knowledge, this is the first study to apply the RMT to the CTS-6 and the BCTQ. The RMT has advantages in assessing measurement constructs and item response categories. The RMT model utilizes predefined mathematics-based criteria into which the data should fit for successful measurement of a latent trait. The generalizability of the present results to other language versions is strong. The RMT assumes that there is one latent trait in the scale. This has its limitations in the analysis, such as when assessing a scale for symptoms that may have multiple aspects. Further research could investigate how the CTS-6 functions in longitudinal settings both as a 6-item scale and as two distinct scales for pain and numbness. This would provide further knowledge on the signal-to-noise ratio for the CTS-6 index score and its scores measuring for two different latent traits.

## Conclusions

By fitting the data to the RMT model, the CTS-6 showed superior psychometric properties compared to its original version, the BCTQ Symptom Severity Scale, in surgically treated carpal tunnel release patients. The CTS-6 might perform more accurately if separated into two sets of items with separate scores measuring pain or numbness as a specific latent trait. The Functional Status Scale of the BCTQ has acceptable structural validity, although several of its items display differential item functioning favoring women or men and different age groups.

## Supplementary information

**Additional file 1.** The residual correlation matrices of the 6-item CTS symptoms scale (CTS-6), Symptom Severity Scale (SSS) and Functional Status Scale (FSS) of the Boston Carpal Tunnel Questionnaire (BCTQ).

## Data Availability

Data is available upon reasonable request to the authors.

## Abbreviations

ACC51: Code for decompression and freeing of adhesion of median nerve
BCTQ: Boston Carpal Tunnel Questionnaire
COSMIN: COnsensus-based Standards for the selection of health Measurement INstruments
CTS-6: Six-item carpal tunnel symptoms scale
DIF: Differential item functioning
ENMG: Electroneuromyography
FSS: Functional status scale
IRT: Item response theory
PRO: Patient-reported outcome
RMT: Rasch Measurement Theory
SSS: Symptom severity scale
viz.: a synonym for the Latin word *videlicet* “that is to say”
